# A novel recombinant serotype 4 fowl adenovirus expressing fiber-2 protein of duck adenovirus 3

**DOI:** 10.3389/fcimb.2023.1177866

**Published:** 2023-03-28

**Authors:** Yiwen Guo, Yun Lin, Quan Xie, Wenyuan Zhang, Zhenqi Xu, Yifei Chao, Xudong Cao, Huiru Jiang, Han Li, Tuofan Li, Zhimin Wan, Hongxia Shao, Aijian Qin, Jianqiang Ye

**Affiliations:** ^1^ Key Laboratory of Jiangsu Preventive Veterinary Medicine, Key Laboratory for Avian Preventive Medicine, Ministry of Education, College of Veterinary Medicine, Yangzhou University, Yangzhou, Jiangsu, China; ^2^ Jiangsu Co-Innovation Center for Prevention and Control of Important Animal Infectious Diseases and Zoonoses, Yangzhou, Jiangsu, China; ^3^ Joint International Research Laboratory of Agriculture and Agri-Product Safety, The Ministry of Education of China, Yangzhou University, Yangzhou, Jiangsu, China; ^4^ Institutes of Agricultural Science and Technology Development, Yangzhou University, Yangzhou, Jiangsu, China

**Keywords:** DAdV-3, fiber-2, recombinant FAdV-4, CRISPR/Cas9, expression, replication

## Abstract

Recently, the highly pathogenic serotype 4 fowl adenovirus (FAdV-4) and duck adenovirus 3 (DAdV-3) were outbroken and widespread, causing substantial economic losses to the duck industry. Therefore, there is an urgent need to generate a recombinant genetic engineering vaccine candidate against both FAdV-4 and DAdV-3. In this study, a novel recombinant FAdV-4 expressing the Fiber-2 protein of DAdV-3, designated as rFAdV-4-Fiber-2/DAdV-3, was generated based on CRISPR/Cas9 and Cre-LoxP systems. Indirect immunofluorescence assay (IFA) and western blot (WB) showed that the Fiber-2 protein of DAdV-3 in rFAdV-4-Fiber-2/DAdV-3 was expressed successfully. Moreover, the growth curve revealed that rFAdV-4-Fiber-2/DAdV-3 replicated efficiently in LMH cells and even showed a stronger replication ability compared to the wild type FAdV-4. The generation of the recombinant rFAdV-4-Fiber-2/DAdV-3 provides a potential vaccine candidate against both FAdV-4 and DAdV-3.

## Introduction

Adenoviruses, belonging to the family *Adenoviridae*, have caused a variety of diseases in poultry and humans (Li et al., 2017). *Adenoviridae* family is currently divided into five genera, including *Mastadenovirus*, *Aviadenovirus*, *Atadenovirus*, *Siadenovirus* and *Ichtadenovirus* (Shah et al., 2017). Adenoviruses are non-enveloped with an icosahedral capsid, and the inner DNA genome is linear, double-stranded, non-segmented and ranged from 26 to 45 kb in size (Davison et al., 2003). The viral particles of adenoviruses consist of some non-structural proteins and three major capsid proteins, containing Hexon, Penton base, and Fiber (Russell, 2009). Hexon, the most abundant component of virus particle, is characterized by specific antigenic determinants (Kulanayake and Tikoo, 2021). Penton acts as a bond to link Fiber and Hexon to stabilize the viral capsid and mediates the attachment and endocytosis of invading virion (Russell, 2009). Fiber is responsible for the interaction with cellular receptors and plays vital roles in viral pathogenicity (Wang et al., 2020).

In 2014, duck adenovirus 3 (DAdV-3) was reported as the aetiological agent of a disease characterized by swollen as well as hemorrhagic liver and kidney, with a mortality rate up to 40% (Wan et al., 2018). Recently, the outbreaks of DAdV-3 have posed a great threat to the duck industry in China (Yin et al., 2022). In 2015, an infectious disease outbroke in commercial duck flocks in Shandong province. The agent mainly infected 25-40 days old ducks and the disease was characterized by typical hydropericardium and hepatitis. According to the later published report, the agent was identified as serotype 4 fowl adenovirus (FAdV-4) (Chen et al., 2017). The prevalence of FAdV-4 in ducks poses a serious challenge to the prevention and control of this disease (Yu et al., 2018). Although several inactivated or subunit vaccines have been developed against FAdV-4 and DAdV-3, there is an urgent need to develop a novel bivalent vaccine candidate against both FAdV-4 and DAdV-3.

The Fiber-2 protein of DAdV-3 can induce neutralizing antibodies and be used as an efficient protective immunogen to offer complete protection against DAdV-3 infection (Yin et al., 2019; Lin et al., 2023). In our previous study, *fiber-2*-edited or *fiber-2*-deleted FAdV-4 is a highly attenuated and protective vaccine candidate (Xie et al., 2021; Xie et al., 2022). In this study, the *fiber-2* gene in FAdV-4 was replaced with that of DAdV-3 using CRISPR/Cas9 and Cre-LoxP systems, and a novel recombinant virus rFAdV-4-Fiber-2/DAdV-3 was generated and characterized.

## Materials and methods

### Cells, viruses, antibodies and plasmids

Leghorn Male Hepatoma cell line (LMH) from the American strain collection center (ATCC) was cultured in Dulbecco Modified Eagle Medium/F12 (Gibco, NY, USA) containing 10% fetal bovine serum (Lonsera, Shanghai, China) at 37°C with 5% CO_2_. The DAdV-3 strain GD and the FAdV-4 strain SD were isolated and stored in our laboratory. Monoclonal antibody (mAb) 3D9 and 5C3 against Fiber-2 of DAdV-3 were prepared and stored in our laboratory. mAb 1B5 against Hexon of FAdV-4 and mAb 1C9 against Fiber-2 of FAdV-4 were prepared and stored in our laboratory. The pMD19-HAL-LoxP-RFP-LoxP-HAR simple vector carrying the RFP expression cassette was constructed and stored in our laboratory. The vector pcDNA3.1-Cre was constructed and stored in our laboratory.

### Construction of sgRNA and donor plasmids

The sgRNA targeting *fiber-2* gene of FAdV-4 was designed through the CRISPR guide RNA designing website (http://zlab.bio/guide-design-resources) and cloned into lentiCRISPR v2 vector. The donor plasmid pMD19-HAL-Fiber-2-LoxP-RFP-LoxP-HAR was constructed based on the linearized plasmid pMD19-HAL-LoxP-RFP-LoxP-HAR simple vector and amplified *fiber-2* gene of DAdV-3 using ClonExpress II One Step Cloning kit (Vazyme, Nanjing, China). The corresponding primers were listed in **Table 1**.

**Table 1:** Primers for construction of the plasmids.

Name	Direction	Sequence (5’-3’)
sgRNA-F	Forward	CACCGAAGGGTGTATCGCTCTCCGG
sgRNA-R	Reverse	AAACCCGGAGAGCGATACACCCTTC
pMD19-Fiber-2-F	Forward	ATCATCATCAAGAACAAACGGACCAACAGATCAGA
pMD19-Fiber-2-R	Reverse	CTTTCTAGACTCGAGCTAATTAACATTTGATGGGTTG
pMD19-linear-F	Forward	CTCGAGTCTAGAAAGCTTGG
pMD19-linear-R	Reverse	GTTCTTGATGATGATGGGAT

### Generation of the recombinant virus rFAdV-4-Fiber-2/DAdV-3-RFP

LMH cells were transfected with 4 µg sgRNA and 4 µg donor plasmid. At 24 h post-transfection (hpt), the LMH cells were infected with FAdV-4 at 0.1 multiplicity of infection (MOI). Then, a fluorescence microscope was used to observe the recombinant virus with RFP. The generated recombinant virus was identified by PCR and purified by limiting the dilution and virus plaque assay. The corresponding primers for the PCR detection of the recombinant virus were listed in **Table 2**.

**Table 2:** Primers for the detection of the recombinant rFAdV-4-Fiber-2/DAdV-3.

Name	Direction	Sequence (5’-3’)
F1-1182-F	Forward	GTTACGTCTACTCCCCCAAC
33288-R	Reverse	CGTTCATGACTCTTTATTTGACACGCGG

### Deletion of the RFP expression cassette in rFAdV-4-Fiber-2/DAdV-3-RFP

LMH cells were transfected with 4 µg pcDNA3.1-Cre. At 2 hpt, rFAdV-4-Fiber-2/DAdV-3-RFP was inoculated into the transfected LMH cells. After 4 days, the supernatant of the infected LMH cells was inoculated into the fresh LMH cells transfected with pcDNA3.1-Cre. The process was repeated until the RFP expression completely disappeared. The new recombinant virus rFAdV-4-Fiber-2/DAdV-3 was identified by indirect immunofluorescence assay (IFA) and western blot (WB).

### Growth curve of the recombinant virus rFAdV-4-Fiber-2/DAdV-3

LMH cells were respectively infected with rFAdV-4-Fiber-2/DAdV-3 and wild type FAdV-4 at 0.1 MOI. During the following 4 days, the supernatants were collected daily and used for virus titration. The TCID_50_ of the supernatants was calculated by the Reed-Muench method (Reed and Muench, 1938) and the viral growth curves of the two viruses were constructed based on GraphPad Prism 8 software.

### The stability of the recombinant virus rFAdV-4-Fiber-2/DAdV-3

The purified recombinant virus rFAdV-4-Fiber-2/DAdV-3 was passaged in LMH cells. The viral supernatants of different passages of rFAdV-4-Fiber-2/DAdV-3 were collected for PCR detection. The corresponding primers were showed in **Table 2**. The LMH cells infected with different passages of rFAdV-4-Fiber-2/DAdV-3 were harvested for detection of Hexon of FAdV-4 and Fiber-2 of DAdV-3 by WB.

### Western blot

LMH cells infected with the recombinant virus were collected and lysed with RIPA buffer (CWbio, Beijing, China) containing protease inhibitors and phosphatase inhibitors (CST, MA, USA). The lysates were boiled for 10 min and centrifuged for 3 min. After SDS-PAGE, the proteins were transferred onto nitrocellulose membrane (GE Healthcare Life sciences, Freiburg, Germany). After blocked with 5% skim milk in PBST for 2 h at room temperature (RT), the membrane was incubated with mAb 5C3 for 2 h at RT. After three washes with PBST, the membrane was incubated with HRP-conjugated goat anti-mouse IgG (Sigma-Aldrich, USA) for 1 h. The dilution ratio of the secondary antibody was 1:10000. After another three washes, the membrane was developed with chemiluminescent reagents and imaged with an automatic imaging system (Tanon 5200).

### Indirect immunofluorescence assay

LMH cells, infected with the recombinant and wild FAdV-4, respectively, were fixed with the prechilled fixative solution (acetone and ethanol with the ratio of 3:2) for 5 min. Then, mAb 3D9 diluted in PBS was incubated with the fixed LMH cells for 45 min at 37°C. After three washes with PBS, the LMH cells were incubated with FITC-conjugated goat anti-mouse IgG (Sigma-Aldrich, USA) for another 45 min. The dilution ratio of the secondary antibody was 1:150. After three washes with PBS, the LMH cells were observed under an inverted fluorescence microscope (Nikon ECLIPSE Ti2, Japan).

## Results

### Generation of the recombinant virus rFAdV-4-Fiber-2/DAdV-3-RFP

**Figure 1:**
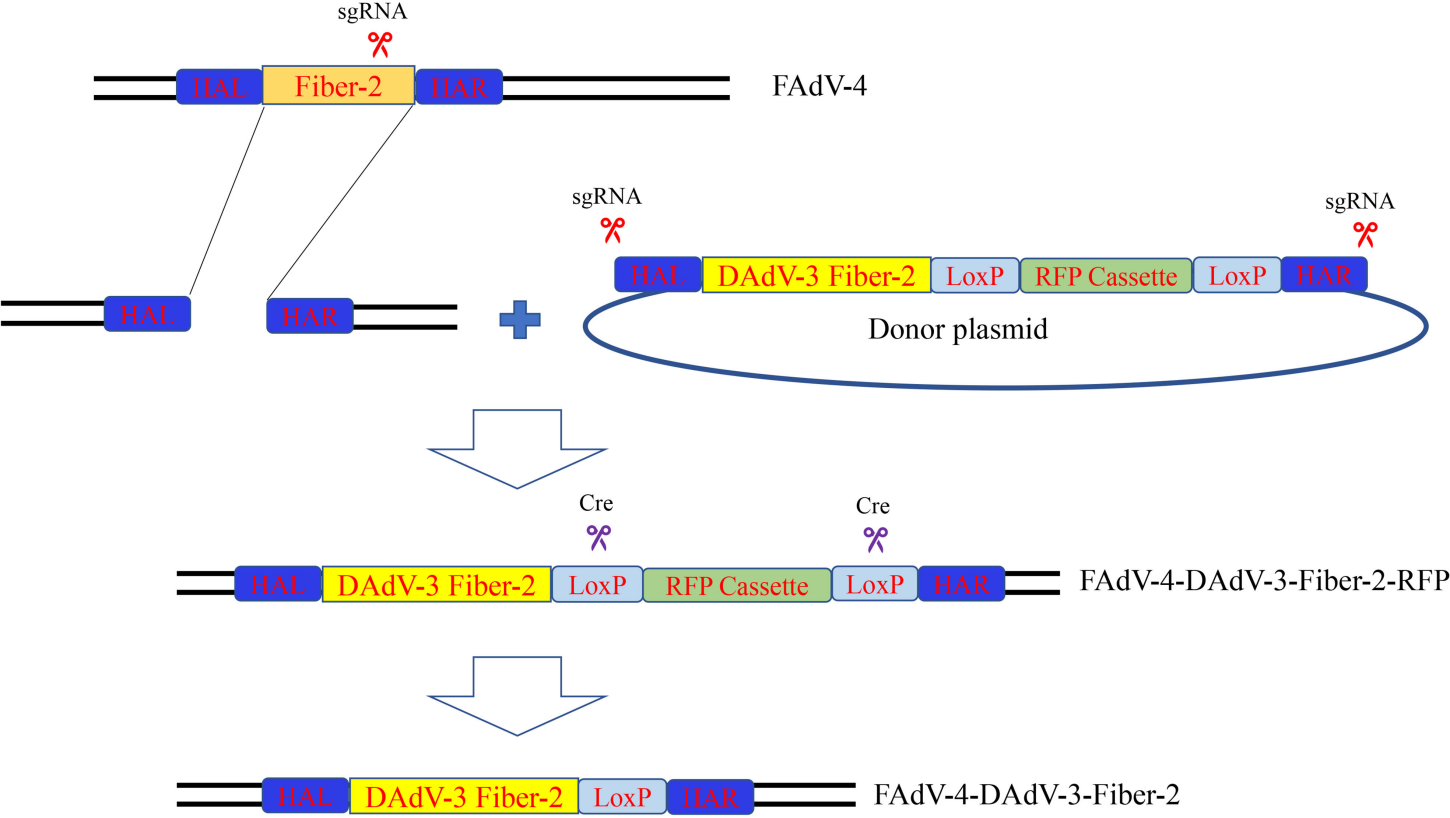
Strategy for the generation of the recombinant virus. LMH cells were transfected with sgRNA and donor plasmid. At 24 hpt, the LMH cells were infected with FAdV-4. The recombinant virus rFAdV-4-Fiber-2/DAdV-3-RFP was purified by limiting dilution assay and viral plaque assay. Fresh LMH cells transfected with pcDNA3.1-Cre were infected with rFAdV-4-Fiber-2/DAdV-3-RFP and the process was repeated until the red fluorescence completely disappeared. The recombinant virus rFAdV-4-Fiber-2/DAdV-3 was finally generated.

**Figure 2:**
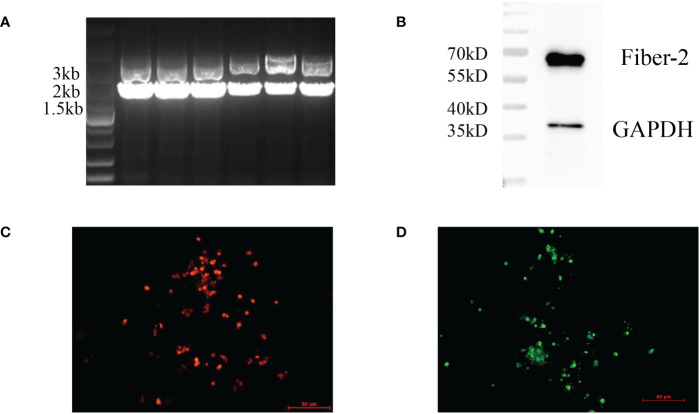
Generation and identification of rFAdV-4-Fiber-2/DAdV-3-RFP. The generation of the recombinant virus rFAdV-4-Fiber-2/DAdV-3-RFP was identified by PCR **(A)**, WB **(B)**, observing the red fluorescence under a fluorescence microscope **(C)**, and IFA **(D)**. The different lanes in the PCR result **(A)** represent the LMH cell samples of different wells on a 96-well plate.

To generate a recombinant FAdV-4 expressing Fiber-2 protein of DAdV-3, CRISPR/Cas9 and Cre-LoxP systems were used in the study and the strategy was showed in **Figure 1**. Firstly, a sgRNA targeting *fiber-2* gene of the FAdV-4 and a donor plasmid were constructed. Then the LMH cells, transfected with sgRNA and donor plasmid for 48 h, were infected with FAdV-4. After the transfection of sgRNA and donor plasmid and the infection of FAdV-4, the recombinant virus rFAdV-4-Fiber-2/DAdV-3-RFP was generated as showed in **Figure 2A**. The PCR result showed that the recombinant virus and the wild type FAdV-4 both present in the LMH cell samples of different wells on a 96-well plate. The red fluorescence under fluorescence microscope displayed in **Figure 2C** also confirmed the generation of rFAdV-4-Fiber-2/DAdV-3-RFP. As described in **Figures 2B, D** using mAbs against Fiber-2 of DAdV-3, the expression of the Fiber-2 protein in the recombinant virus rFAdV-4-Fiber-2/DAdV-3-RFP was confirmed by WB and IFA. The specific fluorescence and band with responding molecular weight for Fiber-2 of DAdV-3 in IFA and WB, respectively, could be detected in LMH cells infected with rFAdV-4-Fiber-2/DAdV-3-RFP. All of these data showed that the novel recombinant virus rFAdV-4-Fiber-2/DAdV-3-RFP expressing the Fiber-2 of DAdV-3 was successfully generated.

### Plaque purification and identification of rFAdV-4-Fiber-2/DAdV-3-RFP

**Figure 3:**
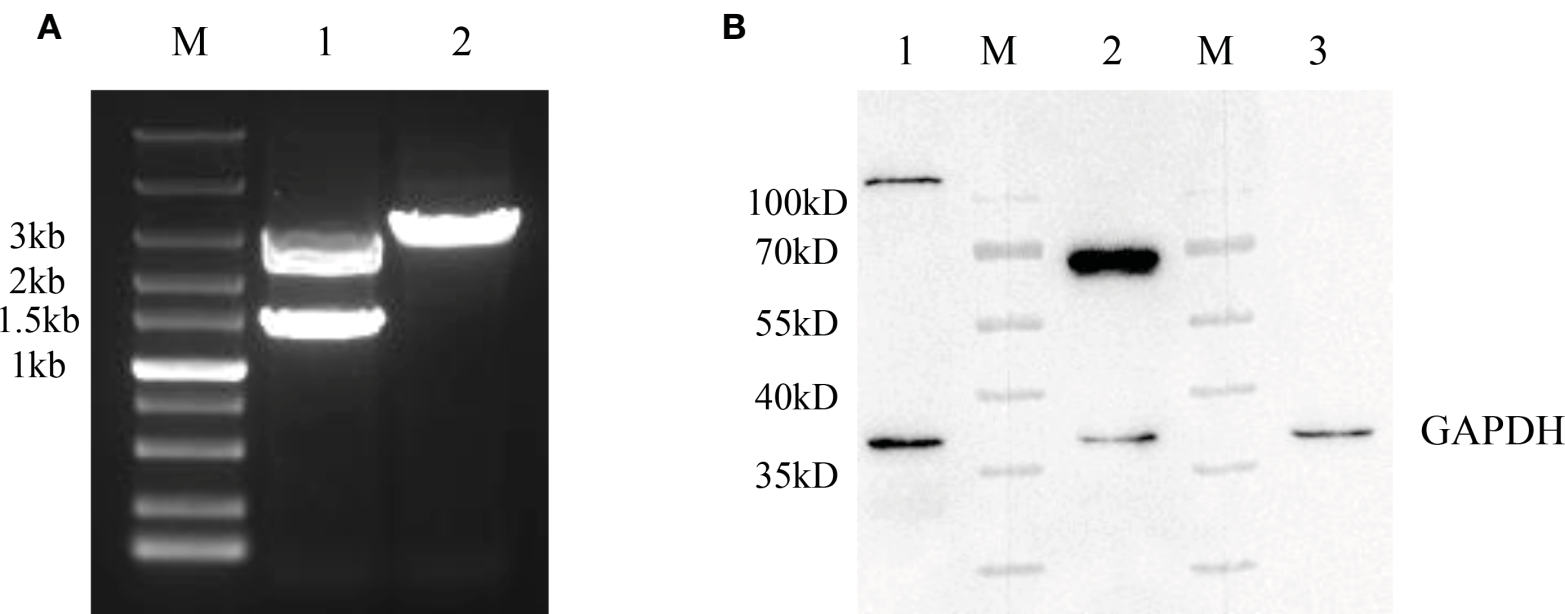
Purification and identification of rFAdV-4-Fiber-2/DAdV-3-RFP. **(A)** The purified recombinant viruses rFAdV-4-Fiber-2/DAdV-3-RFP was identified by PCR. M: Marker; Lane 1: Unpurified rFAdV-4-Fiber-2/DAdV-3-RFP; Lane 2: Purified rFAdV-4-Fiber-2/DAdV-3; **(B)** The purified recombinant virus rFAdV-4-Fiber-2/DAdV-3-RFP was identified by WB. M: Protein marker; Lane 1: Hexon of FAdV-4; Lane 2: Fiber-2 of DAdV-3; Lane 3: Fiber-2 of FAdV-4.

After the generation of rFAdV-4-Fiber-2/DAdV-3-RFP, the recombinant virus was purified by limiting dilution and plaque assay. To identify the purification of the recombinant virus, PCR and WB assays were performed. In **Figure 3A**, a distinct band with corresponding size of molecular weight of about 3000bp (Lane 2) could be amplified in the purified rFAdV-4-Fiber-2/DAdV-3-RFP, whereas two bands for both wild type FAdV-4 and rFAdV-4-Fiber-2/DAdV-3-RFP could be amplified in the unpurified rFAdV-4-Fiber-2/DAdV-3-RFP. As shown in **Figure 3B**, the bands for Hexon protein of FAdV-4 and Fiber-2 protein of DAdV-3 were found, but the band for the Fiber-2 protein of FAdV-4 was not detected in the purified rFAdV-4-Fiber-2/DAdV-3-RFP. All these demonstrated that the recombinant virus rFAdV-4-Fiber-2/DAdV-3-RFP was efficiently purified and without the contamination of wild type FAdV-4.

### Plaque purification and identification of rFAdV-4-Fiber-2/DAdV-3

**Figure 4:**
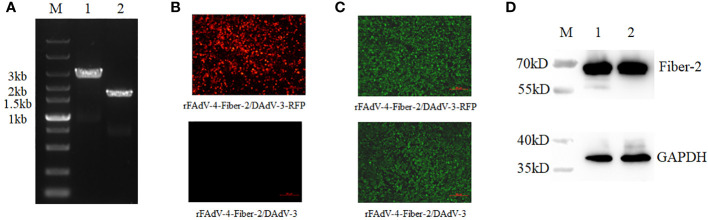
Purification and identification of rFAdV-4-Fiber-2/DAdV-3. **(A)** The purified recombinant virus rFAdV-4-Fiber-2/DAdV-3 was identified by PCR. M: Marker; Lane 1: rFAdV-4-Fiber-2/DAdV-3-RFP; Lane 2: rFAdV-4-Fiber-2/DAdV-3; **(B)** The purified recombinant virus rFAdV-4-Fiber-2/DAdV-3 was identified by observing the red fluorescence under a fluorescence microscope; **(C)** The expression of Fiber-2 protein of DAdV-3 in rFAdV-4-Fiber-2/DAdV-3-RFP and rFAdV-4-Fiber-2/DAdV-3 was identified by IFA; **(D)** The expression of Fiber-2 protein of DAdV-3 in rFAdV-4-Fiber-2/DAdV-3-RFP (Lane 1) and rFAdV-4-Fiber-2/DAdV-3 (Lane 2) was identified by WB.

After the plaque purification of the rFAdV-4-Fiber-2/DAdV-3-RFP, the RFP expression cassette in rFAdV-4-Fiber-2/DAdV-3-RFP was deleted by the Cre recombinase through the transfection of pcDNA3.1-Cre in LMH cells infected with rFAdV-4-Fiber-2/DAdV-3-RFP. As described in **Figure 4A**, a band with corresponding size of molecular weight of about 2000 bp (Lane 2) was displayed in the DNA sample extracted from the purified rFAdV-4-Fiber-2/DAdV-3. The LMH cells infected with rFAdV-4-Fiber-2/DAdV-3-RFP showed lots of red fluorescence under the fluorescence microscope as shown in **Figure 4B**, whereas the LMH cells infected with rFAdV-4-Fiber-2/DAdV-3 did not show any red fluorescence. Moreover, the expression of the Fiber-2 protein of DAdV-3 in rFAdV-4-Fiber-2/DAdV-3 was identified by IFA and WB as described in **Figures 4C, D** by using monoclonal antibody against the Fiber-2 protein of DAdV-3. All these demonstrated that the recombinant virus rFAdV-4-Fiber-2/DAdV-3 expressing the Fiber-2 protein of DAdV-3 was efficiently purified.

### rFAdV-4-Fiber-2/DAdV-3 efficiently replicated *in vitro* with high viral titer

**Figure 5:**
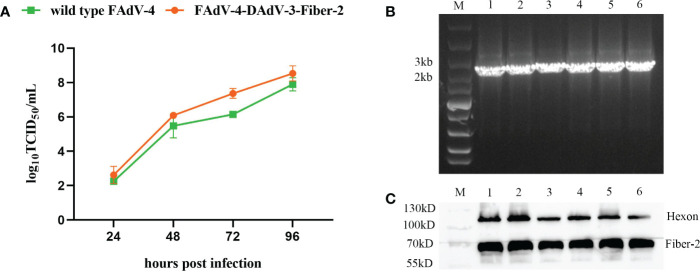
The Growth curve and the stability of the recombinant virus rFAdV-4-Fiber-2/DAdV-3. **(A)** The growth curve of the recombinant virus rFAdV-4-Fiber-2/DAdV-3 was determined by TCID_50_. LMH cells were infected with rFAdV-4-Fiber-2/DAdV-3 and wild type FAdV-4 at 0.1 MOI, respectively. The viral supernatants of the infected LMH cells were collected at the indicated time points and the viral titers were determined by TCID_50_; **(B)** The stability of the recombinant virus rFAdV-4-Fiber-2/DAdV-3 was identified by PCR; **(C)** The stability of the recombinant virus rFAdV-4-Fiber-2/DAdV-3 was identified by WB. The different lanes from 1 to 6 in the PCR result **(B)** and WB result **(C)** represent different passages from 1 to 6.

To investigate the growth characteristics of the purified recombinant virus rFAdV-4-Fiber-2/DAdV-3 *in vitro*, rFAdV-4-Fiber-2/DAdV-3 and wild type FAdV-4 were inoculated into LMH cells at 0.1 MOI, respectively, and the viral supernatants were collected at the indicated time points and the viral titers were determined by TCID_50_. As shown in **Figure 5A**, the recombinant virus rFAdV-4-Fiber-2/DAdV-3 could replicate efficiently in LMH cells with the peak titer of 10^8.5^ TCID_50_/mL, which even showed a stronger replication ability in comparison with the wild type FAdV-4. The high yield of the recombinant rFAdV-4-Fiber-2/DAdV-3 is beneficial to the cost reduction of the potential vaccine candidate against both FAdV-4 and DAdV-3.

### High stability of rFAdV-4-Fiber-2/DAdV-3 *in vitro*

To test the stability of the recombinant virus, rFAdV-4-Fiber-2/DAdV-3 was serially passaged for 6 passages in LMH cells, and the vital supernatants were examined by PCR. As shown in **Figure 5B**, the unique bands specific to rFAdV-4-Fiber-2/DAdV-3 were amplified. Moreover, the infected LMH cells were harvested and used for WB. As shown in **Figure 5C**, the Fiber-2 protein of DAdV-3 and the Hexon protein of FAdV-4 were efficiently expressed in rFAdV-4-Fiber-2/DAdV-3. All these data demonstrate that the rFAdV-4- Fiber-2/DAdV-3 can stably replicate in LMH cells.

## Discussion

Currently, the infection of FAdV-4 has been reported in ducks, including Cherry Valley ducks, Muscovy ducks, and specific pathogen free (SPF) Jinding ducks (Chen et al., 2017; Yu et al., 2018; Wu et al., 2022). The infected ducks were characterized by typical hydropericardium and hepatitis, resulting in great economic losses to the duck industry (Wu et al., 2022). Notably, DAdV-3 infection has become increasingly common in ducks, and its widespread poses great challenges to the duck industry (Shi et al., 2022). To date, some inactivated, recombinant subunit or attenuated vaccines against FAdV-4 or DAdV-3 have been developed. However, a bivalent vaccine against both FAdV-4 and DAdV-3 is not available.

Previous study showed that the Fiber-2 protein of DAdV-3 could not only induce the production of neutralizing antibodies but also provide efficient protection against DAdV-3 infection as a subunit vaccine (Yin et al., 2019; Lin et al., 2023). Our previous studies revealed that Fiber-1 of FAdV-4 directly triggered the viral infection *via* its shaft and knob domains and Fiber-2 of FAdV-4 was identified as a major virulent determiner (Wang et al., 2020). More recently, we found that Fiber-2 of FAdV-4 was not necessary for viral replication and induction of neutralizing antibody, and *fiber-2*-edited or *fiber-2*-deleted FAdV-4 was a highly attenuated and protective vaccine candidate (Xie et al., 2021; Xie et al., 2022), highlighting that *fiber-2* can be as an editable or inserting site for generating live-attenuated recombinant FAdV-4 vaccines against both FAdV-4 and other pathogens. In this study, the *fiber-2* gene in FAdV-4 was replaced with the *fiber-2* gene of DAdV-3 to generate a *fiber-2*-edited recombinant FAdV-4 expressing the *fiber-2* gene of DAdV-3 through CRISPR/Cas9 and Cre-LoxP systems. In this system, the recombinant FAdV-4 with foreign gene rescued in LMH cells would be easily found through the observation of the RPF, which was different from the wild type FAdV-4 without RFP. Through the pickup of the plaque with RPF, the recombinant rFAdV-4-Fiber-2/DAdV-3-RPF could be efficiently purified by limit dilution under fluorescence microscopy. The RPF in the purified recombinant rFAdV-4-Fiber-2/DAdV-3-RPF could be further efficiently deleted by the transfection of Cre recombinase. Notably, rFAdV-4-Fiber-2/DAdV-3 displayed a slightly stronger replication ability compared the wild type FAdV-4 *in vitro* and could reach the titer of 10^8^ TCID_50_/mL in LMH cells. The high yield titer of rFAdV-4-Fiber-2/DAdV-3 satisfied the requirement of mass production and reduced cost for vaccine preparation. It should be also mentioned that two *fiber-2*-edited recombinant FAdV-4 viruses FA4-EGFP and FAdV4-EGFP-rF2 in our laboratory were highly attenuated and could provide efficient protection against wild type FAdV-4 (Xie et al., 2021; Xie et al., 2022). Our preliminary data demonstrated that the inoculation of a high dose of rFAdV-4-Fiber-2/DAdV-3 (2×10^6^ TCID_50_/200ul) in two-week old SPF chickens did not cause any clinical symptoms of the infected chickens and could induce high level of antibody against both FAdV-4 and DAdV-3 with neutralizing titer (Data not shown). Whether rFAdV-4-Fiber-2/DAdV-3 can protect the ducks against both FAdV-4 and DAdV-3 needs to be further evaluated.

In summary, this is the first demonstration of the generation of a novel recombinant virus rFAdV-4-Fiber-2/DAdV-3 expressing the Fiber-2 protein of DAdV-3 using CRISPR/Cas9 and Cre-LoxP systems. The recombinant virus rFAdV-4-Fiber-2/DAdV-3 can not only highly express the Fiber-2 protein of DAdV-3, but also efficiently replicate in LMH cells with high yield, highlighting the value of the recombinant virus as a potential vaccine candidate against both FAdV-4 and DAdV-3.

## Data availability statement

The original contributions presented in the study are included in the article/supplementary material. Further inquiries can be directed to the corresponding authors.

## Author contributions

YG, YL and JY designed the study and analyzed the data. YG, YL and JY wrote the manuscript. YG, YC, WZ, XC, HJ, HL, TL, ZW, QX and ZX performed the experiments. JY, HS and AQ analyzed the data. JY and YL supervised the experiments and acquired the research funds. All authors contributed to the article and approved the submitted version.
